# Spatially resolved transcriptomic signatures of hippocampal subregions and *Arc*-expressing ensembles in active place avoidance memory

**DOI:** 10.3389/fnmol.2024.1386239

**Published:** 2024-10-31

**Authors:** Isaac Vingan, Shwetha Phatarpekar, Victoria Sook Keng Tung, Alejandro Iván Hernández, Oleg V. Evgrafov, Juan Marcos Alarcon

**Affiliations:** ^1^School of Graduates Studies, Program in Neural and Behavioral Sciences, State University of New York, Downstate Health Sciences University, Brooklyn, NY, United States; ^2^Institute for Genomics in Health, State University of New York, Downstate Health Sciences University, Brooklyn, NY, United States; ^3^School of Graduate Studies, Program in Molecular and Cell Biology, State University of New York, Downstate Health Sciences University, Brooklyn, NY, United States; ^4^Department of Pathology, State University of New York, Downstate Health Sciences University, Brooklyn, NY, United States; ^5^The Robert F. Furchgott Center for Neural and Behavioral Science, State University of New York, Downstate Health Sciences University, Brooklyn, NY, United States; ^6^Department of Cell Biology, State University of New York, Downstate Health Sciences University, Brooklyn, NY, United States; ^7^Department of Genetics, Human Genetics Institute of New Jersey, Rutgers University, Piscataway, NJ, United States

**Keywords:** spatial transcriptomics, gene expression, memory, hippocampus, immediate early gene, IEG-expressing ensemble, memory-associated neuronal ensemble

## Abstract

The rodent hippocampus is a spatially organized neuronal network that supports the formation of spatial and episodic memories. We conducted bulk RNA sequencing and spatial transcriptomics experiments to measure gene expression changes in the dorsal hippocampus following the recall of active place avoidance (APA) memory. Through bulk RNA sequencing, we examined the gene expression changes following memory recall across the functionally distinct subregions of the dorsal hippocampus. We found that recall induced differentially expressed genes (DEGs) in the CA1 and CA3 hippocampal subregions were enriched with genes involved in synaptic transmission and synaptic plasticity, while DEGs in the dentate gyrus (DG) were enriched with genes involved in energy balance and ribosomal function. Through spatial transcriptomics, we examined gene expression changes following memory recall across an array of spots encompassing putative memory-associated neuronal ensembles marked by the expression of the IEGs *Arc*, *Egr1*, and *c-Jun*. Within samples from both trained and untrained mice, the subpopulations of spatial transcriptomic spots marked by these IEGs were transcriptomically and spatially distinct from one another. DEGs detected between *Arc* + and *Arc*− spots exclusively in the trained mouse were enriched in several memory-related gene ontology terms, including “regulation of synaptic plasticity” and “memory.” Our results suggest that APA memory recall is supported by regionalized transcriptomic profiles separating the CA1 and CA3 from the DG, transcriptionally and spatially distinct IEG expressing spatial transcriptomic spots, and biological processes related to synaptic plasticity as a defining the difference between *Arc* + and *Arc*− spatial transcriptomic spots.

## 1 Introduction

The neural operations supporting memory require the involvement of various brain systems interconnected through neural networks (Kitamura et al., 2017). The activity of these networks is fine-tuned by experience through the selective recruitment of ensembles of neurons ascribed to contain the bits of information associated with memory (Josselyn and Tonegawa, 2020). Neuronal ensemble activity is shaped by synaptic plasticity mechanisms that modulate the weight and efficacy of the ensemble’s synaptic connections (Takeuchi et al., 2014; Malenka and Bear, 2004). Changes in gene expression are a key underlying mechanism in synaptic plasticity (Mayford et al., 2012), and recent studies have identified multiple profiles of gene expression associated with memory (Marco et al., 2020; Rao-Ruiz et al., 2019; Reshetnikov et al., 2020).

In the rodent hippocampus, encoding of spatial memory information is supported by the diversity of synaptic computations within and across its sub-fields (Poo et al., 2016; Mayford, 2014). Each hippocampal subregion (i.e., Dentate Gyrus, CA1, CA2, CA3) shows distinct patterns of activity to process spatial information and to form or retrieve memories (Middleton and McHugh, 2020; Chevaleyre and Siegelbaum, 2010; Senzai, 2019; Basu and Siegelbaum, 2015; Amaral and Witter, 1989; Sloviter and Lomo, 2012). At the regional level, patterns of activity are conferred through the combination of cyto-architecture, local synaptic circuitry, neuronal cell types and synaptic inputs unique to each region (van Strien et al., 2009; Senzai, 2019; Kesner et al., 2004; Lisman, 1999; Ryan et al., 2015; Hunsaker and Kesner, 2013; Molitor et al., 2021; Rao-Ruiz et al., 2019; Shpokayte et al., 2022; Sullivan et al., 2021; Shah et al., 2016; Cho et al., 2016). At the single-cell level, the functional state of hippocampal neurons is defined by connectivity, hippocampal subregional location, and experience-dependent recruitment into a memory ensemble (Josselyn and Tonegawa, 2020; Tonegawa et al., 2018). The investigation of memory across molecular and functional levels necessitates an approach that connects the scales of hippocampal organization between cellular mechanisms and networks of cells. Though hundreds of individual genes supporting synaptic plasticity at the cellular level have been identified (Sanes and Lichtman, 1999; Alberini and Kandel, 2014; Asok et al., 2019; Magee and Grienberger, 2020; Malenka and Bear, 2004), the transcriptomic profiles which characterize memory across hippocampal subregions and memory-associated neuronal ensembles are understudied.

In this study, we present an exploratory approach to expand our understanding of the spatial organization of transcriptomic profiles by providing evidence that hippocampal subregions undergo distinct differential gene expression of biological mechanisms to support memory. We characterized changes in gene expression across the major subregions of the hippocampus using bulk RNA sequencing of micro-dissected dorsal hippocampal subregions from mice that learned an active place avoidance memory task. The regionalized changes in gene expression with memory in the hippocampus is a developing area of investigation often using behavioral paradigms like fear conditioning or the Morris Water Maze (Cho et al., 2016; Sardoo et al., 2022; Reshetnikov et al., 2020; Harris et al., 2020). Our approach involved training mice in the active place avoidance paradigm (Cimadevilla et al., 2000; Stuchlik et al., 2013), a spatial memory task that distinguishes itself from these other aversive paradigms because it requires proper utilization of cognitive control to solve the spatial task (Chung et al., 2021; Kelemen and Fenton, 2016). Results from our bulk RNA sequencing data suggest that the consolidation of an active place avoidance memory is supported by regionalized transcriptomic profiles separating the CA1 and CA3 from the DG.

Following the establishment of the memory-associated transcriptomic profiles of bulk hippocampal subregions we deepened our exploration of these transcriptomic changes using an innovative RNA sequencing methodology known as spatial transcriptomics. Spatial transcriptomics provides localized whole transcriptome sequencing across tissue sections at near single cell resolution (a.k.a. spatial transcriptomic spots) (Stahl et al., 2016). With this approach, we investigated the putative memory-associated neuronal ensemble by characterizing the spatial distribution of immediate early gene (IEG)-associated gene expression across coronal dorsal hippocampal sections. Memory-associated neuronal ensembles are a sparsely distributed population of neurons which are strongly activated together during the acquisition and recall of a memory, and are thought to encode the traces of information relevant to that memory (Ryan et al., 2015; Guan et al., 2016; Chen et al., 2019). Numerous studies have identified IEGs such as *Arc*, *c-Fos*, *Egr1*, *c-Jun*, *Npas4*, *Bdnf*, and *Fmrp* to characterize memory-associated neuronal ensembles (Sauvage et al., 2019; Josselyn and Tonegawa, 2020; Meenakshi et al., 2021; Erwin et al., 2020). Moreover, the expression of *Arc* mRNA has been used to tag and characterize the properties of memory-associated neuronal ensembles (Guzowski et al., 1999; Ramirez-Amaya et al., 2005; Lee et al., 2022).

Through our combined understanding of IEG activation and memory-associated neuronal ensembles, we used the detection of IEG expression in spatial transcriptomic spots to infer the location of these ensembles and explore the transcriptomic changes supporting spatial memory in the hippocampus. Consistent with our bulk RNA sequencing findings, spatial transcriptomics also showed differential gene expression between the hippocampal regions from an active place avoidance memory trained compared to an untrained mouse. We then investigated the difference between memory-associated neuronal populations through the analysis of spatial transcriptomic spots expressing the IEGs *Arc*, *Egr1*, and *c-Jun*. Comparison of the gene expression profiles from these IEG-expressing spatial transcriptomic spots between the memory trained and the untrained mouse revealed differential expression of genes involved in energy production and cytoplasmic translation. Novel analyses comparing IEG*-*expressing spatial transcriptomic spots *within* each mouse hippocampal sample (i.e., within the memory trained mouse or within the untrained mouse) revealed that IEG-expressing spatial spots were transcriptomically and spatially distinct from one another. With *Arc*, only the comparison between *Arc-*expressing and non-*Arc*-expressing spatial transcriptomic spots in the memory trained mouse –but not the untrained mouse– detected gene expression profiles linked with synaptic plasticity and memory. Additionally, similar comparisons amongst *Egr1* and *c-Jun* expressing versus non-expressing spots revealed collections of differentially expressed distinguishing each of the investigated populations of IEG-expressing spots within the trained sample.

While still at an exploratory level, our spatial transcriptomics results highlight the role of *Arc* in shaping memory-associated ensembles and of the detection of gene expression profiles unique to the comparison *Arc-expressing* versus non-*Arc*-expressing spatial transcriptomic spots in an animal trained in the active place avoidance memory task. We speculate that a neuronal network being actively recruited in the consolidation of memory (such as the hippocampal network in our study) manifests transcriptomically divergent cellular microenvironments (such as the *Arc-expressing* and non-*Arc*-expressing spatial transcriptomic spots in our study) that could facilitate the processing of memory information.

## 2 Methods

### 2.1 Animals

A total of 18 adult ArcCreERT2::eYFPflx mice (Denny et al., 2014) with C57BL/6 genetic background aged 3–4 months were utilized across all experimental cohorts. Bulk RNA sequencing experiments utilized 3 male and 3 female mice, 2 females and 1 male were assigned to the trained behavioral condition, and 1 female and 2 males were assigned to the untrained behavioral condition (see section “Behavior” for details). Spatial transcriptomics experiments utilized 2 male mice, 1 mouse was assigned to each behavioral condition. RT-qPCR experiments utilized 12 male mice, with 6 mice assigned to each behavioral condition. Prior to experimental onset mice were bred in-house at the SUNY Downstate Health Sciences University vivarium (Brooklyn, NY, USA). Mice were housed in groups of two to five per cage. Beginning on the first day of training and testing, mice were single housed in shoebox cages in a sound attenuation cubicle (Med Associates). For every experimental run, a minimum of two mice —each undergoing the same behavioral conditioning— were simultaneously housed in the sound attenuation cubicle. *Ad libitum* food and water was provided. Mice were randomly assigned to behavioral cohorts before the start of the experiment. Mice were handled daily for 3 days prior to the start of the experiment to reduce anxiety and improve voluntary approach. All animal procedures proposed are approved by, and will be performed following, the Institutional Animal Care and Use Committee guidelines at SUNY Downstate Health Sciences University.

### 2.2 Behavior

All procedures were performed in compliance with the Institutional Animal Care and Use Committee of the State University of New York, Downstate Health Sciences University. ArcCreERT2::eYFPflx male mice were trained in a hippocampus-dependent two-frame active place avoidance task. The place avoidance system consisted of a 40-cm diameter arena with a parallel rod floor that could rotate at 1 rpm. The position of the animal was tracked using PC-based software (Tracker, Bio-Signal Group Corp., Brooklyn, NY) that analyzed 30-Hz digital video images from an overhead camera. Mice in the trained condition learned the active place avoidance task. Place avoidance of a 60° zone was reinforced by a constant current foot shock (60 Hz, 500 ms, 0.2 mA) that was scrambled (5-poles) across pairs of the floor rods. Rotation of the arena would carry the mouse into the shock zone unless the animal actively avoided the zone. Entering the shock zone for more than 500 ms triggered shock. Additional shocks occurred every 0.5 s until the animal left the shock zone. Measures of place avoidance were computed by TrackAnalysis software (Bio-Signal Group Corp., Brooklyn, NY, USA). The number of detections of the animal in the shock zone was tracked as the performance metric for the training trials.

On day one, mice assigned to the trained behavioral condition (trained mice) received a 30-min trial with the shock off to habituate to the rotating arena. Across the next 2 days the animals experienced four training trials, with two 30-min trials a day with the activated shock with a 40 min inter-trial-interval. Control (untrained mice) experienced identical training conditions, except for the shock always being off. The number of detections of the animal in the shock zone was tracked as the training performance metric for the training trials. Memory retention performance was assessed 24 h after the final training session in a 10-min retention test with the shock off. Latency to the animal’s second entrance into the shock zone was tracked as the memory retention performance metric for the retention test. Previous research indicates the latency to second entrance as a valid behavioral metric for retention test performance because it represents place avoidance memory recall that is less affected by to the animal’s potential errors in self-localization once placed in the rotating arena (Cimadevilla et al., 2000; Stuchlik et al., 2013; Cimadevilla et al., 2001).

### 2.3 Microdissected bulk RNA sequencing

60 min post retention test, mice were euthanized (5% isoflurane vaporized in 100% oxygen), and brains were extracted, washed in ice cold artificial cerebrospinal fluid, blocked, and mounted on a vibratome stage. Hippocampal subregions (DG, CA3, CA1) were microdissected from 400 μm thick coronal live tissue sections. Subregions from the dorsal hippocampus were extracted and microdissected using microsurgical tools. Microdissected pieces of tissue from each animal were pooled by subregion in 500 μL of prechilled TRIzol in a 1.5 mL microcentrifuge tubes and stored at −80°C.

Total RNA was extracted using the Direct-zol RNA miniprep kit (Zymo Research, Irvine, CA, United States) according to the manufacturer’s protocol. RNA quality was assessed on an Agilent 2200 TapeStation (Agilent Technologies, Palo Alto, CA, United States). Samples with a RIN greater than 7 were processed for library preparation with NEBnext rRNA depletion kit and ULTRAII FS RNA-seq Library Preparation Kit for Illumina (New England Biolabs, Ipswich, MA, United States). This procedure involved steps for mRNA enrichment, fragmentation, random primed cDNA synthesis, second strand synthesis, end repair, A-tailing, adaptor ligation, and PCR amplification. Size selection and cleanup was performed with SPRI select beads (Beckman Coulter, Indianapolis, IN, United States). Sequencing was performed on the NovaSeq 6000 (Illumina, Inc., San Diego, CA, United States) to obtain paired end sequencing reads.

### 2.4 Bulk RNA sequencing data preprocessing

RNA sequencing reads obtained from NovaSeq 6000 were systemically processed to ensure high-quality outputs for downstream analyses. To evaluate the quality of the reads, quality control analysis was performed using *FASTQC* v0.11.9. RNA sequencing reads in Binary Base Call (BCL) format were subsequently converted using *bcl2fastq2* v2.20 to FASTQ format, and simultaneously demultiplexing to assign sequences to their respective samples based on index sequences. Demultiplexed samples that did not pass quality control were excluded from future analyses in the pipeline. Next, FASTQ files were then mapped to mouse MM10 (GRCm39) reference genome using *STAR* 2.7.10a aligner. Lastly, the transcript quantification of the reads was done using *Salmon* v1.2.0 to create a count matrix for downstream analyses.

### 2.5 Bulk RNA sequencing analyses

We conducted several differential expression analyses using *DESeq2* v1.1.0 in the Illumina BaseSpace, comparing RNA counts between trained and untrained sample groups. Genes with a false discovery rate (FDR) below 0.05 were deemed differentially expressed. To identify the variances in our count data, we performed principal component analysis (PCA) on the normalized count data, encompassing all hippocampal regions and within each specific region.

To visualize our data, we generated a heatmap in *ComplexHeatmap* v2.18.0 using Z-scores, which standardized the expression levels of genes across samples (Gu et al., 2016). The heatmap, organized with dendrogram and sorted using Euclidean distance, allowed us to identify patterns of similarity in gene expression across samples.

To explore deeper into biological significance, we conducted Gene Ontology (GO) enrichment analysis using *clusterProfiler* v4.10.0 in R (Wu et al., 2021), focusing on biological processes, cellular components, and molecular functions. Only enriched GO terms with an FDR less than 0.01 were identified as significant, and the top 10 GO terms were displayed in a ranked dot plot. Redundant terms were filtered out using *simplify* with a cutoff of 0.7. Biological processes were ordered along the *y*-axis based on their ordered significance within each column from left to right. Additionally, if a biological process in one analysis is amongst the top 10 of another analysis, the dot signifying its enrichment will be shown at the corresponding row in the plot.

We also utilized *Vennplex* v1.0.0.2 software to compare and visualize the overlaps among differentially expressed genes (DEGs) from our differential expression analyses (Cai et al., 2013). The gene lists were categorized into upregulated and downregulated, allowing the visualizing of the gene overlaps and identification of counter-regulated genes (genes exhibiting opposite expression trends in different regions). We employed the *SuperExactTest* v1.1.0 in R to statistically assess whether the observed overlapping genes across different hippocampal regions significantly exceeded the expected overlap by random coincidence (Wang et al., 2015). The assessment was performed using hypergeometric distribution and Fisher’s Exact test, and the statistical significance of these gene overlaps was shown in upset-style plots.

### 2.6 Tissue preparation for spatial transcriptomics

60 min after retention test, mice were euthanized and brains were extracted and immediately prepared for snap freezing in cuvettes of Tissue-Tek Optimal Cutting Temperature compound (Sakura Finetek USA, Torrance, CA, United States) floating in a bath of methyl-butane chilled by liquid nitrogen. Brain blocks were cryosectioned at −20°C (10X Genomics, 2023) and 10 μm thick coronal sections containing the dorsal portion of the hippocampus were obtained (bregma −1.7 and − 2.2 mm, Paxinos and Franklin, 2019). Collected tissue sections are trimmed to fit within the 5 mm × 5 mm capture area on the Visium gene expression slide. Sections were mounted on a prechilled Visium gene expression slide or a Tissue Optimization slide.

For Optimization, all tissue sections were collected from a single tissue. For Visium gene expression, slide capture areas contained one section per biological sample. Slides were fixed in prechilled methanol before staining using the Visium spatial tissue optimization protocol (Genomics, 2019) or the Visium spatial gene expression protocol (Genomics, 2023).

Tissues permeabilization time on the gene expression slide was set to 18 min based on our tissue optimization results. Images were taken according to the Visium Spatial Gene Expression Imaging Guidelines (Genomics, 2021). Brightfield histology images for the Visium Tissue Optimization and Gene Expression slides were taken using the Leica Aperio CS2 Slide Scanner at 20x magnification. Tissue optimization fluorescent images were taken on a Zeiss LSM 800 (555 nm LED, 75% intensity, and 200 ms exposure).

mRNA was extracted and libraries were prepared following the Visium Spatial Gene Expression User Guide (Genomics, 2023). Libraries were indexed using the Dual Index Kit TT Set A (PN-1000215). Indexed libraries were loaded at 300 pM and sequenced on a NovaSeq 6000 System (Illumina) using a NovaSeq S4 Reagent Kit (200 cycles, catalog no. 20027466, Illumina), targeting a sequencing depth of approximately 2.0 × 10^8^ read-pairs per sample. Sequencing depth was determined through a calculation of a suggestion 50,000 read pairs per spatial capture spot (Genomics, 2023). Our tissue samples covered roughly 80% of the 5,000 spatial capture spots within the Visium fiducial frame [(0.8 × 5,000) × 50,000 = 2.0 × 10^8^]. Sequencing was performed using the following read protocol: read 1: 28 cycles; i7 index read: 10 cycles; i5 index read: 10 cycles; and read 2: 91 cycles.

### 2.7 Spatial transcriptomics preprocessing

Raw RNA sequencing data obtained from NovaSeq 6000 were systemically processed to ensure high-quality outputs for downstream analyses. Raw sequencing data in BCL format were converted using *bcl2fastq2* v2.20 to FASTQ format, and simultaneously demultiplexing to assign sequences to their respective samples based on both i5 and i7 index sequences. Following the conversion, these FASTQ files were subsequently processed with *TrimGalore* v.0.6.5 to automate quality, adapter trimming and perform quality control.

For the alignment and the quantification of gene expression, *Space Ranger* v2.0.0 (10x Genomics) was used to generate and quantify raw gene-barcode matrices from RNA sequencing data. Alignment and quantification of Visium spatial gene expression data utilizes the same tools implemented in the analysis of single cell RNA-seq data sets. Data were aligned to refdata-gex-mm10-2020-A, a mouse genome index provided by 10x Genomics, which is an annotated version of mm10 genome assembly. The gene-barcode matrices identified the number of unique molecular identifiers (UMIs) associated with each gene for each cellular barcode, allowing the quantification of transcript abundance.

After the alignment and quantification, Space Ranger toolset was utilized to normalize the gene expression data within the raw gene-barcode matrices to account for any technical variations. Finally, we also utilized Space Ranger to align gene expression data with spatial coordinates derived from histological images of each sample, facilitating the visualization of transcriptional activity to specific tissue location.

### 2.8 Spatial transcriptomics analysis

Gene expression outputs from Space Ranger were analyzed using the software package *Seurat* v5 in the R coding environment (Hao et al., 2023; Butler et al., 2018). Spatial gene expression data were cropped to only include the capture spots within the dorsal hippocampus. Using default Seurat commands, data from each sample were normalized, transformed (using *SCTransform v2*) and integrated. The integrated data set were further normalized and clustered (resolution = 0.8) in PCA space. Clustered data can be visualized through UMAP projections of the capture spots or in the 2D space of the tissue section. Cell type annotation was performed by integrating our data with a random subset of 10,000 hippocampal cells from the Allen Brain Atlas Cortex and Hippocampus Single Cell Taxonomy (Yao et al., 2021). Predicted cell-type identities were calculated and optimized based on gene expression similarity and anatomical location.

Capture spots were grouped along two different criteria to calculate differential gene expression in our samples. First, capture spots belonging to the whole hippocampus and each anatomical cell layer in the DG, CA3 and CA1 regions were compared across training conditions. Next, within each training condition, hippocampal capture spots were separated into two groups based on the detectable (>0) expression of the immediate early gene (e.g., *Arc +* and *Arc*− groups). IEG expressing and not expressing groups of spots were compared both within training conditions and across training conditions. Differential gene expression was calculated using the Wilcoxon rank sum test (the default method for Seurat). Genes with an of FDR below 0.05 were considered differentially expressed genes/transcripts. GO term enrichment and overlap analyses on detected DEGs were performed as described above for bulk RNA sequencing DEGs.

Expression data for 23 IEGs from all hippocampal spots evaluated through pairwise Pearson’s Correlations using the R-language base stats package. Pearson’s correlation coefficients between all IEG pairs were calculated within samples, alongside *p* values to assess significance. Heatmaps of the correlation matrix were plotted using the R-language package, ComplexHeatmap.

### 2.9 RT-qPCR

Dorsal hippocampal subregions (CA1, CA3, and DG) were microdissected as described for bulk sequencing. Tissue was homogenized using TRIzol (Thermo Fisher Scientific, Waltham, MA, United States) before RNA extraction by guanidinium-phenol-chloroform extraction with ethanol precipitation was performed and quantified using spectrophotometry. cDNA was reverse transcribed from sample bulk RNA using Thermo Fisher’s SuperScript IV Kit (Thermo Fisher Scientific, Waltham, MA, United States). Relative gene expression was quantified using the SYBR green master mix system (Ponchel et al., 2003). 10 μL reactions were prepared in the wells of a 384 well plate, hippocampal regions were each prepared on separated plates. Each well contained 2.5 ng of template cDNA and 0.5 μM each of forward and reverse primers for one gene per well for a total reaction volume of 10 μL (sequences and melting temperatures can be found in Table 1). Each gene was loaded in triplicate in each plate to account for pipetting errors. Plates were incubated in the Bio-Rad CFX384 Real-Time PCR Detection System (Bio-Rad Life Sciences, Hercules, CA, United States) using the following schedule: 50°C for 5 min (1 cycle), 95°C for 10 min (1 cycle), 60°C for 1 min (1 cycle), and 95°C for 15 s followed by 60°C for 1 min (40 cycles).

**Table 1:** RT-qPCR primer sequences.

Gene	Primer	Sequence	TM (°C)
*Bok*	F	AGG TAG TGT CCC TGT ATT CCG	60.4
	R	AAG GTC TTG CGT ACA AAC TCC	60.2
*Crym*	F	GGG AGT CAT GCC TGC CTA C	61.8
	R	AGC CAT TGC TGG GAT CAA AGA	61.8
*Gapdh*	F	AGG TCG GTG TGA ACG GAT TG	57.47
	R	TGT AGA CCA TGT AGT TGA GTC A	52.84
*Homer3*	F	AGG GAA CAG CCA ATC TTC AGC	62.4
	R	GAC ACG GTA AGT GCG TGC T	62.6
*Kit*	F	GCC ACG TCT CAG CCA TCT G	58.51
	R	GTC GCC AGC TTC AAC TAT TAA CT	55.14
*Pcsk1n*	F	GCT GCT GTG CCT AAT ACC CA	60.11
	R	GGA GTG CTC GTC TCA ACC AA	59.97
*Rasal1*	F	GCC AAG GAC GTG TCT GGA AG	62.8
	R	TGA ACG GTG TAC TCC TCC CC	62.8
*Serpina3n*	F	TGT CTG CGA AAC TGT ACC CT	58.95
	R	ACC CAC AGA CAG GCT CAA TG	59.96
*Shank3*	F	TCT TAG CCT TTG ATG CTC CCC	59.79
	R	CAC AGT GTA GTG GCG GAA GA	59.68

Primers were selected to validate the most significant genes with the highest fold change of expression for each regional comparison of trained and untrained animals. The genes *Crym* and *Kit* were selected from the DEGs detected in the DG comparison. *Rasal1* and *Pcsk1n* were selected from the DEGs detected in the CA3 comparison. And *Bok, Homer3, Serpina3n,* and *Shank3* were selected from the DEGs detected in the CA1 comparison.

Ct values for each reaction are automatically determined (roughly 20% of the plateau value). Data were analyzed for relative gene expression quantification utilizing the ∆∆Ct method, using the expression of *Gapdh* as the internal control. Relative expression values were normalized to the expression value of the untrained group. The Levene’s test was uses to assess the homogeneity of variances for Log_2_ transformed relative expression. Upon confirmation of homogeneity of variances, Student’s t-tests were performed on each primer target across training conditions using log_2_ transformed relative expression value for each comparison.

## 3 Results

### 3.1 APA trained mice exhibit place avoidance memory

**Figure 1:**
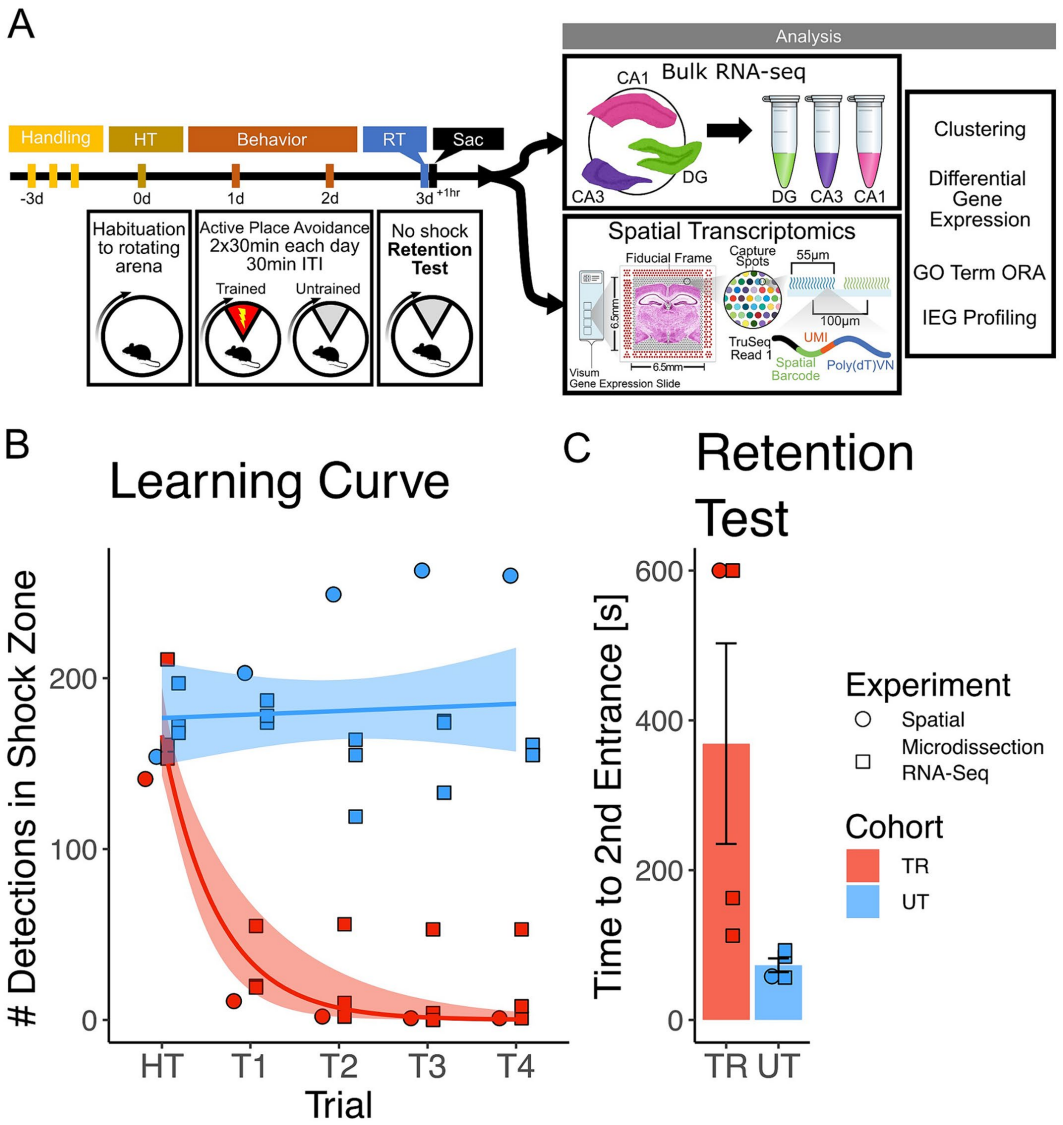
Mice trained in the APA learned to avoid the location of an unmarked shock zone. **(A)** Experimental timeline. Mice were trained in the active place avoidance paradigm over the course of 4 days following 3 days of handling. On day 0 mice were habituated (HT) to a rotating arena with no active shock zone. On day 1 and 2 mice assigned to the trained (TR) behavioral condition (*n* = 4) received two 30-min trials with an active shock zone. Mice assigned to the untrained (UT) behavioral condition (*n* = 4) were not exposed to an active shock zone. On day 3 all mice received a 10-min retention test trial (exposed to the rotating arena with no active shock zone). Mice were sacrificed 60 min following the end of the retention test, brains were collected for further processing. Samples from three of the four trained mice (2 females, 1 male) and untrained mice (1 female, 2 males) were processed with bulk RNA sequencing, while the remaining mice (1 trained male, 1 untrained male) had their samples processed with spatial transcriptomics. **(B)** Training performance measured as number detections in the shock zone. Mice assigned to the trained behavioral condition learned to avoid the location of the shock zone indicated by the decreased number of shocked triggered. Exponential fit learning curves included with shaded error bars (SE). **(C)** Memory performance measured as time to second entrance in the retention test trial. Trained mice demonstrated higher time to second entrance during the retention test. Trained mean 375 s ± 70.4 s (SE), Untrained mean 58.7 s ± 8.51 s (SE). Line and point color reflect the assigned behavioral condition (untrained = blue, trained = red). Point shape reflects the experimental pipeline a given animal was assigned to (square = bulk RNA-seq, circle = spatial transcriptomics).

To investigate gene expression changes induced by active place avoidance (APA) training in the hippocampal network, we conducted bulk RNA-sequencing and spatial transcriptomics (see Figure 1A for experimental timeline). In the APA, mice learn to avoid a 60^°^ shock zone on a rotating circular arena (Cimadevilla et al., 2000; Stuchlik et al., 2013). Mice trained in the APA task with the shock zone on (*n* = 4, 2 females, 2 male) exhibited a decrease in the number of detections in the shock zone during the acquisition trials (Figure 1B) and longer re-entry times to the shock zone during the memory retention test trials (Figure 1C) relative to the mice in the untrained cohort (*n* = 4, 1 female, 3 males). Re-entry times to the shock zone (i.e., time to second entrance) was used as the retention test performance metric because it represents performance that is more resistant to the animal’s potential errors in self-localization once placed in the rotating arena (Cimadevilla et al., 2000; Stuchlik et al., 2013; Cimadevilla et al., 2001). From this pool of animals 3 trained (2 females, 1 male) and 3 untrained (1 female, 2 male) were utilized in Bulk RNA-sequencing experiments and 1 trained and 1 untrained animal (2 males) were utilized in spatial transcriptomic experiments.

### 3.2 Hippocampal subregional transcriptomes are stratified by training condition

**Figure 2:**
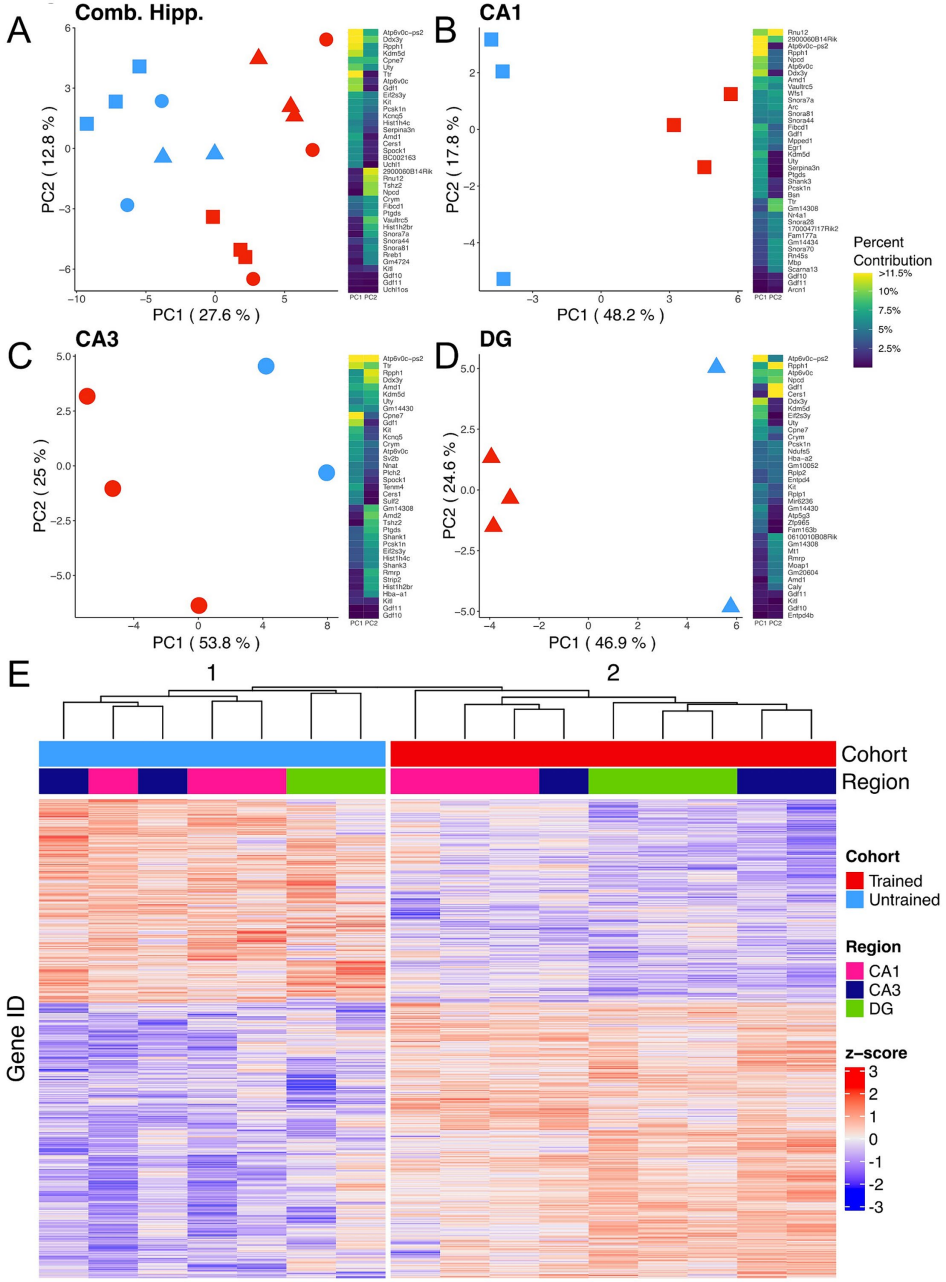
APA training results in substantial changes in hippocampal subregion gene expression profiles. **(A–D)** Left, principal component analyses of all hippocampal subregions **(A)**, CA1 **(B)**, CA3 **(C)**, DG **(D)** illustrate a clear separation between samples collected from trained (red) and untrained (blue) mice along the axis that explains the most variance. Hippocampal regions are also indicated by the point shape, squares refer to the CA1, circles to the CA3, and triangles to the DG. Right, heatmap with top 20 contributing genes to the first two principal components of each are reported in the heat map to the right of each plot. Sample size for the CA1 was 3 trained and 3 untrained. Sample size for the CA3 was 3 trained and 2 untrained. And Sample size for the DG was 3 trained and 2 untrained. Two samples from separate untrained animals (1 DG, 1 CA3) did not pass sequencing quality control and were excluded from PCA analyses. **(E)** Normalized gene expression of the top 2000 most significant DEGs. Hierarchical clustering of gene expression profiles plotted as a dendrogram clusters samples by training condition.

To explore the molecular mechanisms following memory retention test across the dorsal hippocampus, bulk RNA-sequencing was performed on the Dentate Gyrus (DG), CA3, and CA1 hippocampal subregions collected from trained and untrained mice (trained: 2 females, 1 male; untrained: 1 female, 2 male). Principal component analysis (PCA) from all 3 subregions combined reveals a separation of samples by behavioral cohort (Figure 2A), indicating that differences in training contribute substantially to the overall variability of gene expression in our samples. Separation between samples is further accentuated when PCA is performed separately on data from each subregion (Figures 2B–D). Consistently, hierarchical clustering of gene expression profiles reliably grouped samples by their behavioral cohort (Figure 2E).

### 3.3 Hippocampal subregional enrichment of learning and memory-related biological processes

**Figure 3:**
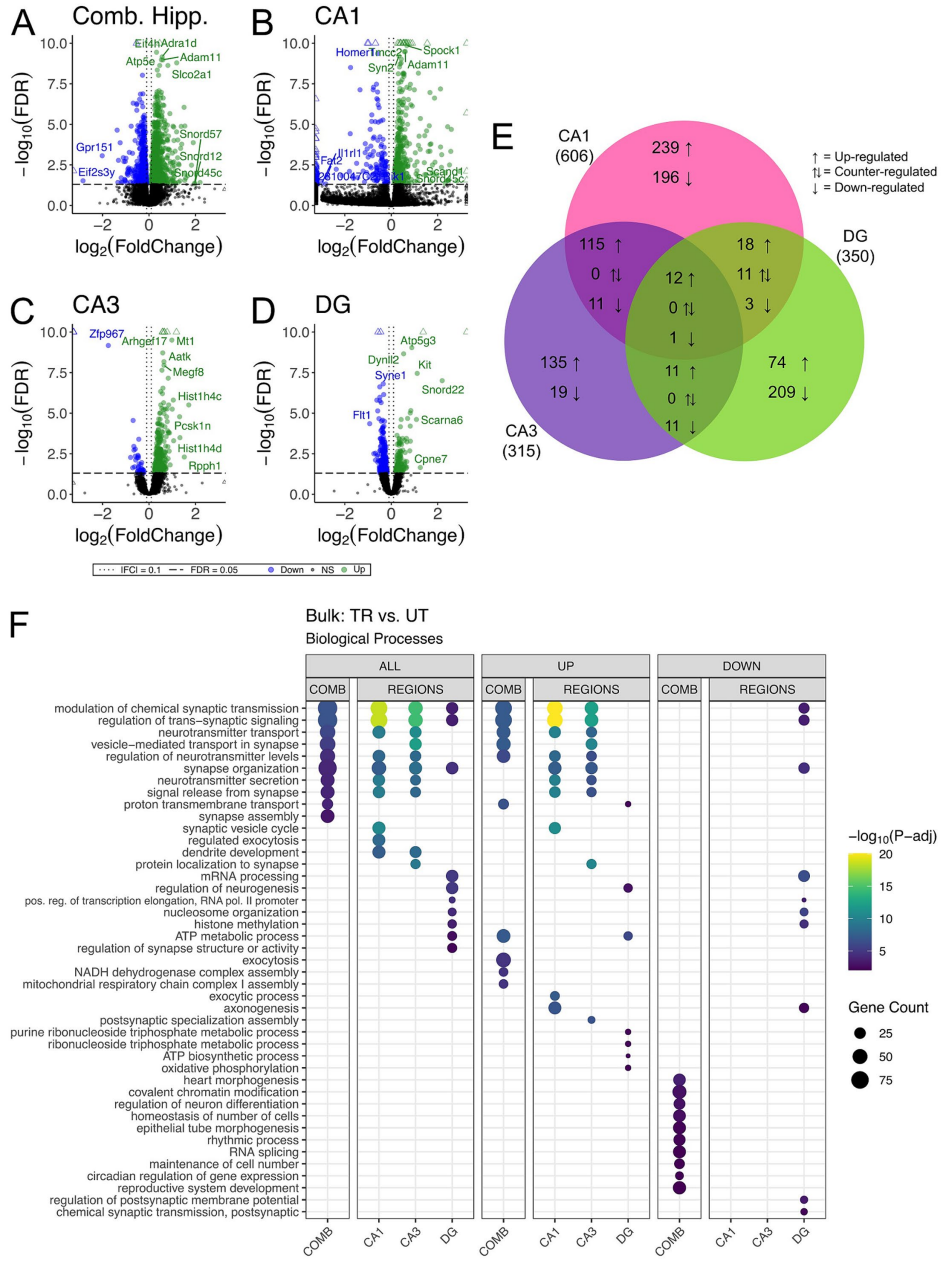
Bulk RNA-seq of hippocampal subregions reveals similar expression profiles in CA1 and CA3 regions in trained samples. **(A–D)** Volcano plot of DEGs between trained and untrained animals in all hippocampal subregions combined **(A)**, and in the CA1 **(B)**, CA3 **(C)**, and DG **(D)** subregions individually. Sample size for the CA1 was 3 trained and 3 untrained. Sample size for the CA3 was 3 trained and 2 untrained. And Sample size for the DG was 3 trained and 2 untrained. Two samples from separate untrained animals (one DG, one CA3) did not pass sequencing quality control and were excluded from differential gene expression analyses. In all hippocampal subregions combined, 966 genes were upregulated and 697 were downregulated. 393 genes were upregulated and 213 were downregulated in the CA1, 273 genes were upregulated and 42 were downregulated in the CA3, and 117 genes were upregulated and 223 were downregulated in the DG. See Supplementary Figures 1A–D for full sized plots. **(E)** Overlaps in regional DEGs demonstrate greatest similarity between CA1 and CA3 subregions. DEGs from each regional analysis were stratified by direction of fold change. Overlaps in DEGs between the CA3 and CA1 subregions were significant (Fisher’s Exact Test). See Supplementary Figure 1E for further details. **(F)** Regional enrichment of biological processes detected amongst all DEGs (left) and stratified by up- (middle) and down-regulated (right) DEGs. The top 10 biological processes detected in each analysis are shown on the *y*-axis. Biological processes are ordered based on their statistical significance in the left-most column in which they are detected. Dot color reflects the statistical significance [−log_10_(FDR)] of the biological process enrichment. Dot size reflects the number of detected DEGs mapped to the genes involved in a given biological process.

We carried out differential gene expression analysis comparing APA-trained to untrained mice. We detected 1,663, 606, 315, and 350 differentially expressed genes (DEGs) (adjusted *p*-value <0.05) in the combined hippocampal subfields, CA1, CA3 and DG respectively, when contrasting APA-trained and untrained mice (Figures 3A–D and Supplementary Figures 1A–D). Despite high statistical significance (FDR < 0.005), most DEG in each trained versus untrained comparison exhibited modest fold change (|log_2_ fold| < 1.0) difference. Next, we investigated the overlap of regionally detected DEGs (FDR < 0.05) to elucidate the distribution of DEGs across hippocampal subregions. We found the largest overlap of upregulated DEGs between the CA3 and CA1 subregions with a total of 127 intersecting genes (Figure 3E and Supplementary Figure 1E).

GO enrichment analysis was conducted to identify overrepresented biological process among DEGs from the trained versus untrained comparison and identified within each hippocampal subregion (The Gene Ontology Consortium, 2019). Among the DEGs detected in the combined hippocampal subregions, we found overrepresentation of genes involved in the modulation of neurotransmitter signaling (Figure 3F). These DEGs included *Ywhag*, *Bsn*, and *Nup153*, which have been previously implicated in these cellular functions (Wachi et al., 2017; Annamneedi et al., 2021; Leone et al., 2019). DEGs detected in both the CA3 and CA1 were also enriched with genes involved in the modulation of neurotransmitter signaling. The genes *Snap25, Shank1, Shank 3, Bsn and Ywhag* were identified in the overlap of upregulated DEGs between these regions and have been studied for their involvement in synaptic transmission (Irfan et al., 2019; Mao et al., 2015; Shi et al., 2017; Kouser et al., 2013). DEGs detected in the DG were enriched with genes involved in neurogenesis and transcriptional regulation. Among these DEGs were *Kit*, *H2afz*, and *Kmt2e*, which have been studied for their relationship to neurogenesis and transcriptional regulation (Katafuchi et al., 2000; Ferreira et al., 2016; Barbieri et al., 2018; Zovkic et al., 2014; Collins et al., 2019). When enrichment analyses were separated by up- and down-regulated DEGs, the differences separating the CA1 and CA3 from the DG became more striking with no significant enrichment of biological processes among the downregulated DEGs of the CA1 and CA3. These data reveal a spatial distribution of gene expression across the dorsal hippocampus of trained animals, defined by a greater degree of similarity between the CA1 and CA3 than between either of these subregions and the DG.

### 3.4 RT-qPCR validation of regionally detected DEGs

We performed targeted gene expression analyses using RT-qPCR on DG, CA3, and CA1 subregion samples collected from similarly behaviorally conditioned (i.e., trained and untrained) animals (Supplementary Figures 3A,B). We probed a total of 8 genes (with *Gapdh* as an internal control), each identified for their high statistical significance and relatively large positive fold change of expression in one of the region-specific comparisons between trained and untrained samples. In the DG, we identified significant differential expression of the gene *Kit* between trained and untrained animals, which replicates our results from bulk RNA sequencing comparisons of in this region (Supplementary Figure 3C). In the CA3 we identified significant differential expression of the gene *Rasal1* between trained and untrained animals, replicating our results from bulk RNA sequencing (Supplementary Figure 3D). In the CA1, *Homer3* and *Crym* were significantly differentially expressed (Supplementary Figure 3E). Differential expression of *Homer3* in the CA1 was an expected validation of our bulk RNA sequencing findings, but the detection of *Crym* differential expression was unexpected as it was selected as a candidate DEG from the DG. In sum, RT-qPCR allowed us to validate the differential expression of select target genes with the largest expression fold changes in our bulk RNA-seq dataset. Consistent with prior reports, some of our target genes which exhibited comparatively lower fold changes of expression escaped detection because of the limitation in RT-qPCR relative to RNA-sequencing approaches (Coenye, 2021; Everaert et al., 2017; Reshetnikov et al., 2020).

### 3.5 Differences in the spatial distribution of hippocampal gene expression between trained and untrained animals

**Figure 4:**
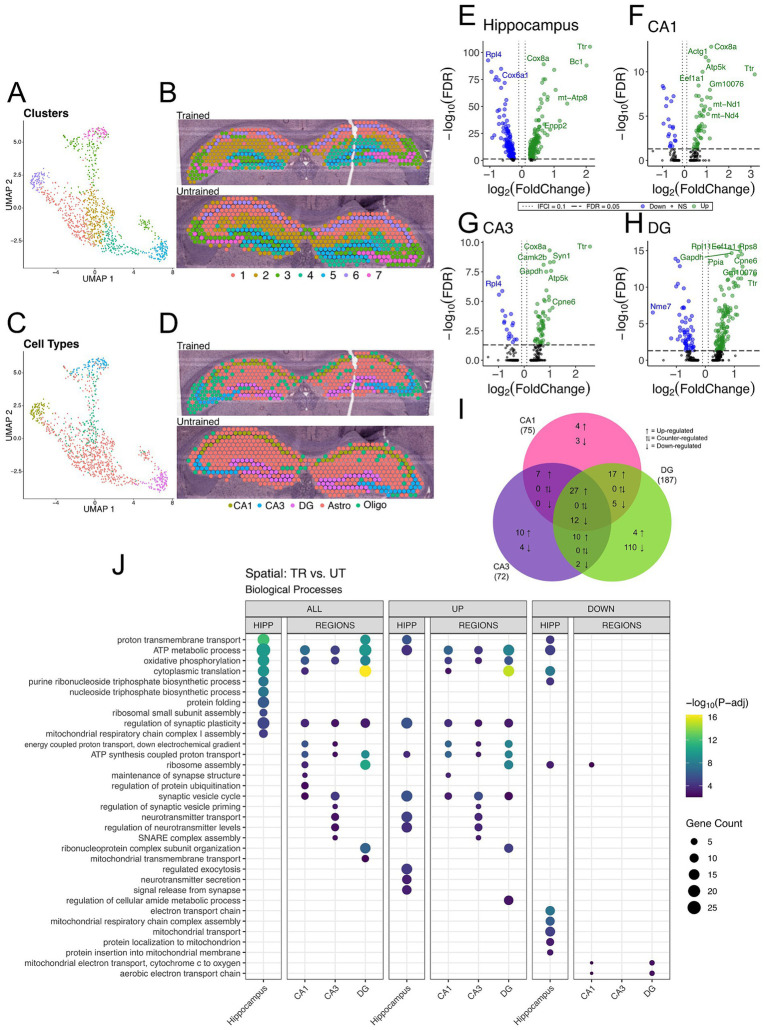
DEGs detected between trained and untrained animals within hippocampal subregions using spatial transcriptomics are enriched with genes involved in similar biological processes to those seen in bulk RNA-seq. **(A,B)** UMAP plot **(A)** and spatial transcriptomic maps of hippocampal spots **(B)** from trained and untrained mouse samples (*n* = 1 per group). Discrete color scale displays unsupervised graph-based clustering of hippocampal spots. Clusters are labeled 1 through 7 from greatest (1) to least (7) number of spots belonging to each unbiased cluster. Unbiased clustering groups spots along anatomical boundaries reflecting natural transcriptomic differences between morphologically distinct regions in the hippocampus. **(C,D)** UMAP plot **(C)** and cell-type annotated spatial transcriptomic maps of hippocampal spots **(D)** in samples from the trained and untrained mouse. Discrete color scale reflects the predominant cell type surveyed by each capture spot which was determined by cell-type annotation of hippocampal capture spots through automated integration of our data with the single cell Allen Brain Atlas for mice. **(E–H)** Volcano plot of DEGs between all hippocampal **(E)**, CA1 **(F)**, CA3 **(G)**, and DG **(H)** cell-layer spots from trained and untrained mice (*n* = 1 per group). In the analysis of all hippocampal combined, 205 genes were upregulated and 147 were downregulated. 55 genes were upregulated and 20 were downregulated in the CA1 cell layer, 54 genes were upregulated and 18 were downregulated in the CA3 cell layer, and 129 genes were upregulated and 58 were downregulated in the DG cell layer. **(I)** Overlap of regionally detected DEGs stratified by direction of fold change. Overlaps were tested with Fisher’s exact test. See Supplementary Figure 2 for further details. **(J)** Regional enrichment of biological processes detected amongst all DEGs (left) and stratified by up- (middle) and down-regulated (right) DEGs. The top 10 biological processes detected in each analysis are shown on the *y*-axis. Biological processes are ordered based on their statistical significance in the left-most column in which they are detected. Dot color reflects the statistical significance [−log_10_(FDR)] of the biological process enrichment. Dot size reflects the number of detected DEGs mapped to the genes involved in a given biological process. See Supplementary Figure 2 for enrichment of Cellular Component and Molecular Function GO terms.

To further investigate the spatial distribution of DEGs associated with APA memory training across the hippocampus, we performed spatial transcriptomics on coronal sections containing the dorsal hippocampus from one trained (male) and one untrained (male) mouse. Computational analyses of integrated capture spots in the hippocampal slices from trained and untrained animals revealed distinct clusters (seen in a Uniform Manifold Approximation and Projection (UMAP) plot) which map along anatomical boundaries in a spatial transcriptomic plot (Figures 4A,B). Spatial transcriptomics data were integrated with the Allen Brain Atlas (Yao et al., 2021) to computationally annotate each spot with the cell-type most prominently detected (Figures 4C,D).

Differential gene expression analysis between trained and untrained mice identified a total of 352 DEGs in all hippocampal spots, with further stratification by subregional cell layers detecting 75 DEGs in the CA1, 72 DEGs in the CA3, and 187 DEGs in the DG (Figures 4E–I and Supplementary Figure 2). GO enrichment analyses of the DEGs detected between all hippocampal spots identified biological processes related to energy production and protein expression (Figure 4J). DEGs detected in the analysis of the CA1 cell layer were enriched with biological processes related to energy production and synaptic function. In the CA3, DEGs were enriched with genes involved in neurotransmitter release and energy production. And in the DG, DEGs were enriched with genes involved in energy production and translational machinery. Notably, the biological process “regulation of synaptic plasticity” (GO: 0048167) was enriched among the DEGs detected between all subregional comparisons between trained and untrained animals.

These spatial transcriptomics data are concordant with our bulk RNA sequencing data. The overlap of DEGs detected in each hippocampal region between the two techniques was lower than expected (10, 10, 22, in the CA1, CA3, and DG, respectively). Notwithstanding, there is stronger concordance between the biological process enrichment detected with the two techniques. We found upregulation of genes involved in the regulation of synaptic plasticity across all three hippocampal subregions in both experiments. These findings reinforce a subregion-specific model of memory-associated transcriptional activation following memory recall.

### 3.6 Arc-expressing hippocampal spots exhibit changes in the expression of memory-associated genes and biological processes

Our data suggest that differences in behavioral conditioning contributed to variations in the spatial distribution of synaptic plasticity related gene expression across the hippocampal network. It is well established that a surge of Arc expression accompanies memory associated processes such as consolidation and retrieval, and differences in behavioral conditioning affect the recruitment of *Arc*-expressing hippocampal neurons which form the memory-associated neuronal ensemble (Gouty-Colomer et al., 2016; Gallo et al., 2018; Carrillo-Reid, 2022; Gouty-Colomer et al., 2016; Hsieh et al., 2021; Chia and Otto, 2013). As such, we studied the *Arc*-expressing spatial transcriptomics spots to assess changes in biological processes within the memory associated neuronal ensemble following APA memory recall. Spatial transcriptomic spots with detectable expression of *Arc* mRNA (>0 copies) were determined to be *Arc +* in this experiment. Given the resolution and capture efficiency of spatial transcriptomics (Asp et al., 2020), these spots represent regions of space with high expression of *Arc* mRNA across one or more cells/cellular compartments. Hence, we utilized these *Arc +* spots as a proxy for the location of the putative memory-associated neuronal populations with induced expression of *Arc* following the retrieval of the active place avoidance memory.

**Figure 5:**
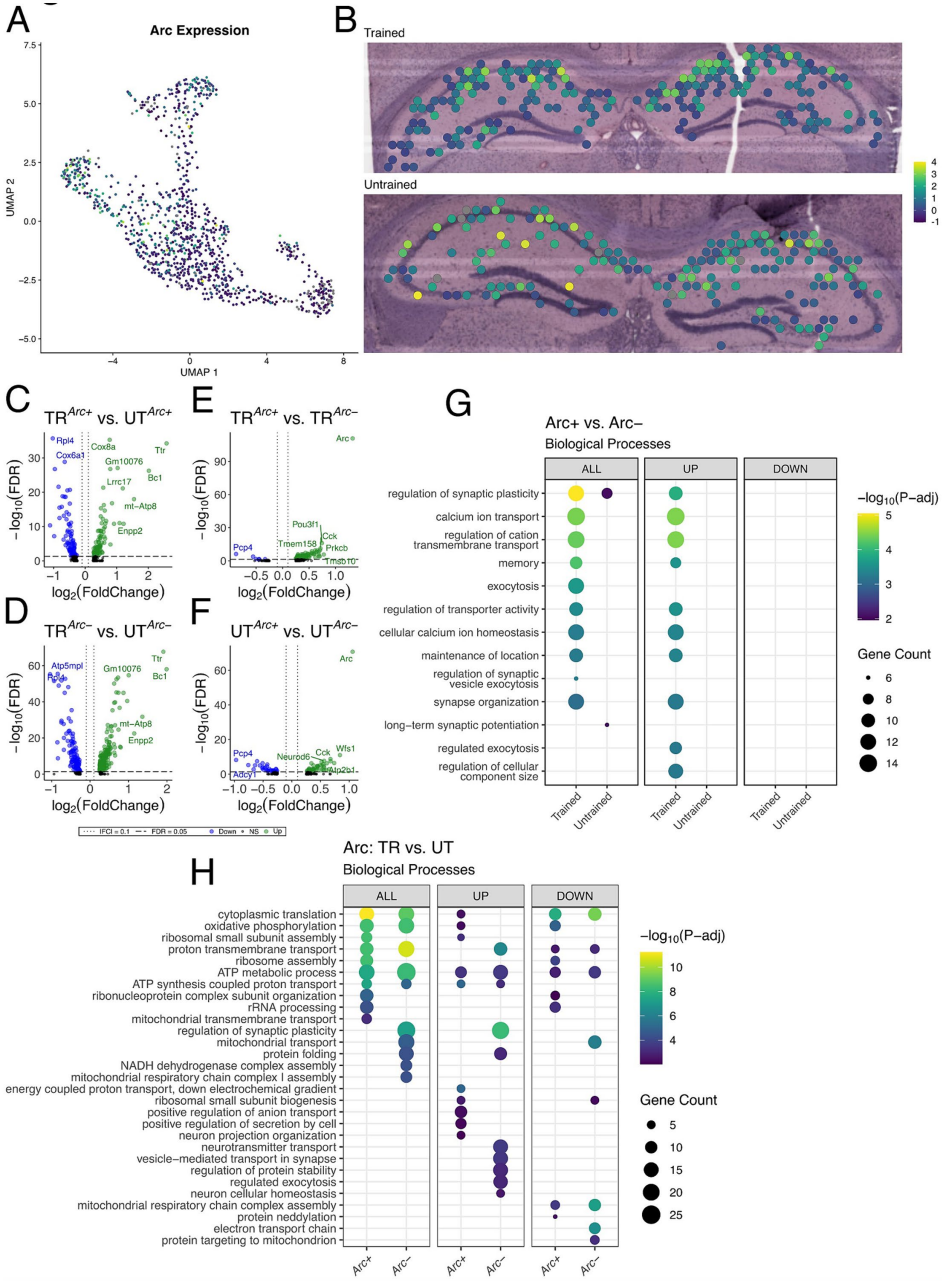
Arc-positive spots in the trained sample are enriched with genes involved in synaptic plasticity. **(A,B)** UMAP plot **(A)** and spatial transcriptomic maps of hippocampal spots **(B)** in the trained and untrained samples colored to reflect normalized expression of *Arc*. *Arc* positivity is determined by detectable expression (>0 copies) of *Arc* mRNA in a spatial transcriptomic spot. **(C,D)** Volcano plots of differential gene expression of *Arc* positive **(C)** and negative **(D)** spots across behavioral conditions. In the analysis of *Arc +* spots across behavioral conditions 85 upregulated and 89 downregulated genes were detected. In the analysis of *Arc-* spots across behavioral conditions 113 upregulated and 240 downregulated genes were detected. **(E,F)** Volcano plots of differential gene expression between *Arc* positive and negative spots within trained **(E)** and untrained **(F)** samples. In the analysis of *Arc* spots within the trained animal 95 upregulated and 6 downregulated genes were detected. In the analysis of *Arc* spots within the untrained animal 47 upregulated and 31 downregulated genes were detected. **(G)** Biological processes overrepresented in the analysis of *Arc*-expressing spots within trained and untrained samples amongst all DEGs (left) and stratified by up- (middle) and down-regulated (right) DEGs. The top 10 biological processes detected in each analysis are shown on the *y*-axis. Biological processes are ordered based on their statistical significance in the left-most column in which they are detected. Dot color reflects the statistical significance [−log_10_(FDR)] of the biological process enrichment. Dot size reflects the number of detected DEGs mapped to the genes involved in a given biological process. **(H)** Biological processes overrepresented in the analysis of *Arc*-expressing spots across trained and untrained samples amongst all DEGs (left) and stratified by up- (middle) and down-regulated (right) DEGs. The top 10 biological processes detected in each analysis are shown on the *y*-axis. Biological processes are ordered based on their statistical significance in the left-most column in which they are detected.

We observed a sparse distribution of *Arc +* spots in our samples consistent with the characteristic expression of Arc protein in the hippocampus from studies investigating memory associated ensembles (Mayford, 2014; Chia and Otto, 2013; Gouty-Colomer et al., 2016). Out of the 542 spots in the hippocampus from the trained animal, 195 spots had detectable expression of *Arc* mRNA (*Arc+*). The untrained animal, 169 *Arc +* spots out of 568 hippocampal spots (Supplementary Figure 4C). In both samples, spots with the highest level of *Arc* expression concentrated primarily in the CA1 cell layer (Figures 5A,B).

To assess synaptic plasticity-related transcriptional changes in these *Arc +* spots, we conducted multiple differential expression comparisons: (1) across sets of *Arc +* or *Arc*− spots between the trained and untrained animal (Figures 5C,D), and (2) within the trained or untrained animal between sets of *Arc +* and *Arc*− (Figures 5E,F). The comparison of *Arc +* spots across trained and untrained animals detected 174 significant DEGs, and comparison of *Arc*− spots across trained and untrained animals detected 353 significant DEGs. GO term enrichment analysis of DEGs detected across *Arc* + spots in the trained and untrained animal revealed enrichment with biological processes related to ribosomal function and energy production (Figure 5H). A finding which is consistent with the regionalized differences observed between the trained and untrained animals through spatial transcriptomics (Figure 4J). *Rpl4* and *Rpl5* were differentially expressed in the analysis across *Arc* + spots in the trained and untrained, and are known for their roles in ribosomal biogenesis (Robledo et al., 2008). Enrichment analyses of DEGs detected in *Arc*− spots across training conditions revealed a similar set biological processes related to energy production as those detected in the analysis of *Arc +* spots. Differences between the two comparisons are accentuated when enrichment analyses are performed separately on up- and down-regulated DEGs.

101 DEGs were detected within *Arc* + and *Arc*− spots in the trained animal, and 78 DEGs were detected within *Arc* + and *Arc*− spots in the untrained animal. Notably, substantially more biological processes related to synaptic plasticity and neuronal excitability were enriched in the DEGs *Arc +* spots of the trained animal as compared to *Arc +* spots of the untrained animal. This difference is enhanced when the enrichment analyses are separated by up- and down-regulated DEGs, where only the upregulated DEGs within the *Arc +* spots of the trained animal demonstrate enrichment of any biological processes (Figure 5G). *Prkcb* and *Dkk3*, known for their role in synaptic plasticity (Weeber et al., 2000; Martin-Flores et al., 2024), were detected amongst upregulated genes in the comparison of *Arc +* and *Arc*− spots in the trained animal. In summary, enrichment analyses in *Arc +* spots across training conditions detected biological processes related to energy metabolism and ribosomal biogenesis, while *Arc +* spots within the trained sample revealed biological processes related to synaptic plasticity and transmission.

### 3.7 IEG-expressing hippocampal spots comprise distinct gene expression profiles

Our findings demonstrate that behavioral training induces discrete transcriptional changes in the population of spots marked by *Arc* expression. However, memory traces are likely comprise overlapping yet molecularly distinct neuronal populations tagged by IEGs (Sun et al., 2020; Nambu et al., 2022; Okuno, 2011; Gallo et al., 2018). We sought to examine spots marked by the expression of the IEG most and least correlated with the expression of *Arc* to evaluate the functional relationship between these subsets of the memory-associated ensemble. Pairwise correlations identified *Egr1* and *c-Jun* as the IEGs most and least correlated with *Arc*, respectively (Supplementary Figures 4A,B, 5A–F).

Unlike *Arc*, which codes for a cytoskeleton regulating protein, both *Egr1* and *c-Jun* code for transcription factors. IEGs which function as transcription factors are thought to promote the expression of activity related genes which execute the cellular level changes necessary for memory storage (Yap and Greenberg, 2018; Sun et al., 2020; Kim et al., 2018; Yelhekar et al., 2024). Genes downstream of *Egr1*-driven transcription are involved in vesicular release, neurotransmitter metabolism, receptor expression, and synaptic plasticity (Duclot and Kabbaj, 2017; Sekiya et al., 2019). Genes downstream of *c-Jun*-driven transcription in the brain are involved in neurite growth, axonal regeneration, and synaptic long term depression (Raivich and Behrens, 2006; Haeusgen et al., 2009).

**Figure 6:**
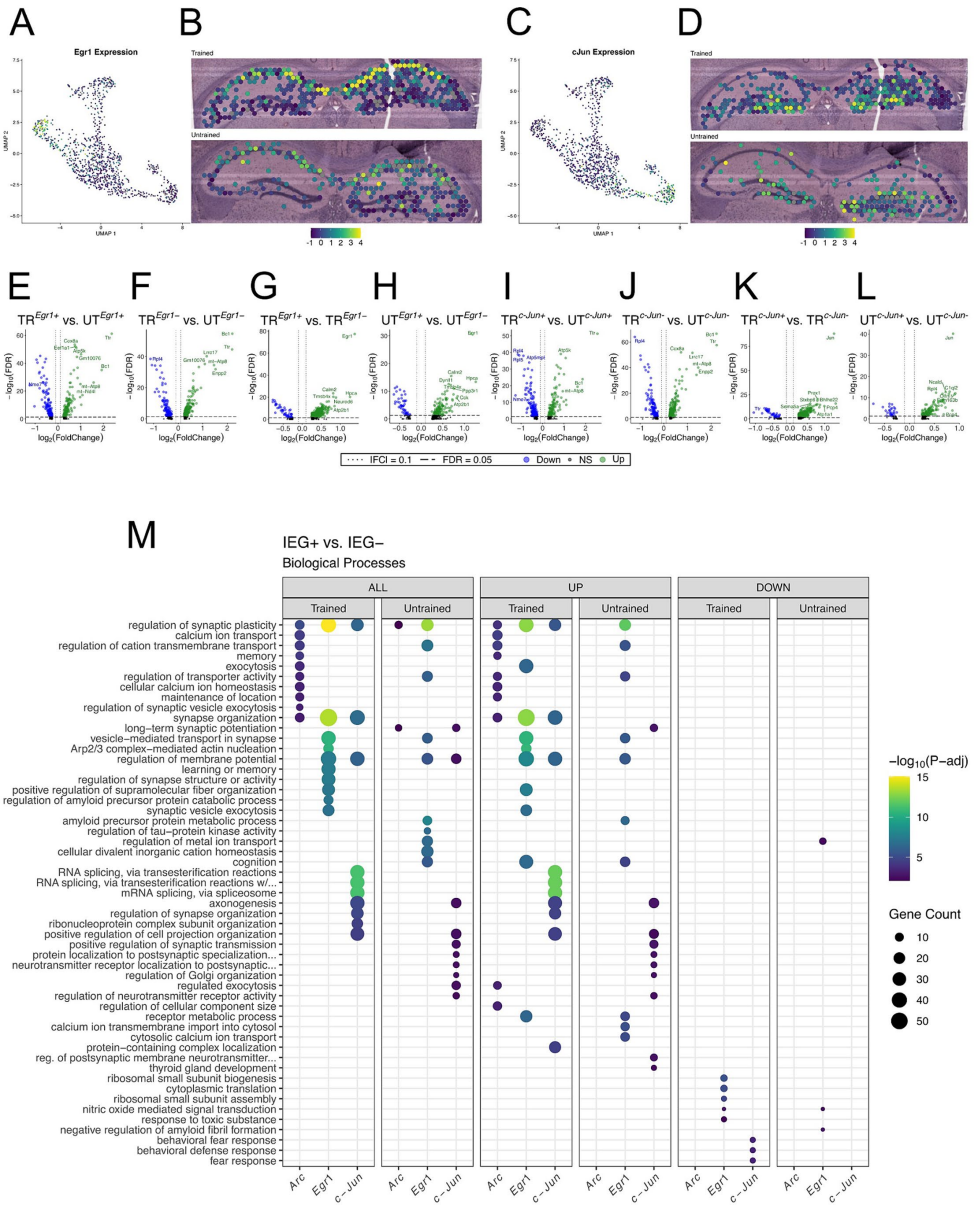
Spatial transcriptomics reveals distinct collections of biological processes enriched among *Arc*, *Egr1*, and *c-Jun*-expressing spots. **(A,B)** UMAP plot **(A)** and spatial transcriptomic maps **(B)** of hippocampal spots in the trained and untrained sample colored to reflect the normalized expression of *Egr1*. **(C,D)** UMAP plot **(C)** and spatial transcriptomic maps **(D)** of hippocampal spots in the trained and untrained samples colored to reflect normalized expression of *c-Jun*. *Egr1* and *c-Jun* positivity is determined by detectable expression (>0 copies) of *Egr1* or *c-Jun* mRNA in a spatial transcriptomic spot. **(E–H)** Volcano plots of differential gene expression of *Egr1* positive **(E)** and negative **(F)** spots across behavioral conditions and within trained **(G)** and untrained **(H)** samples. In the analysis of *Egr1+* spots across behavioral conditions 135 upregulated and 160 downregulated genes were detected. In the analysis of *Egr1-* spots across behavioral conditions 216 upregulated and 111 downregulated genes were detected. In the analysis of *Egr1* spots within the trained animal 509 upregulated and 46 downregulated genes were detected. In the analysis of *Egr1* spots within the untrained animal 150 upregulated and 46 downregulated genes were detected. **(I–L)** Volcano plots of differential gene expression of *c-Jun* positive **(I)** and negative **(J)** spots across behavioral conditions and within trained **(K)** and untrained **(L)** samples. In the analysis of *c-Jun +* spots across behavioral conditions 123 upregulated and 175 downregulated genes were detected. In the analysis of *c-Jun-* spots across behavioral conditions 193 upregulated and 121 downregulated genes were detected. In the analysis of *c-Jun* spots within the trained animal 440 upregulated and 40 downregulated genes were detected. In the analysis of *c-Jun* spots within the untrained animal 138 upregulated and 28 downregulated genes were detected. **(M)** Biological processes overrepresented between the IEG+ and IEG- spots within each sample. Analysis of GO term overrepresentation is stratified by all DEGs (left), up- (middle), and down-regulated (right) DEGs. The top 10 biological processes detected in each analysis are shown on the *y*-axis. Biological processes are ordered based on their statistical significance in the left-most column in which they are detected. Dot color reflects statistical significance [−log10(FDR)] of biological process enrichment. Dot size reflects number of detected DEGs mapped to the genes involved in a given biological process.

We performed differential gene expression analyses on *Egr1* and *c-Jun* expressing spots across and within behavioral conditions. As described in the previous section, spatial transcriptomic spots were determined as positive if they had detectable expression of the IEG (either *Egr1* or *c-Jun*). In total, 380 out of 542 spots in the hippocampus of the trained animal and 256 out of 568 spots in the untrained animal were found to express detectable levels of *Egr1* (Supplementary Figure 3G). In both samples, spots with the highest level of *Egr1* expression were located predominantly in the CA1 cell layer, following the spatial distribution of *Arc* expression (Figures 6A,B). Detectable levels of *c-Jun* were seen in 308 out of 542 spots in the hippocampal sample from the trained animal and 175 out of 568 spots in the untrained animal (Supplementary Figure 3G). In both samples, spots with the highest level of *c-Jun* expression were located predominantly in the DG granule cell layer (Figures 6C,D).

In the analysis of *Egr1+* or *Egr1-* spots across training conditions, 295 DEGs were detected in the *Egr1+* spots, and 327 DEGs in the *Egr-* spots (Figures 6E,F). In the analysis of *c-Jun +* or *c-Jun-* spots across training conditions, 298 DEGs were detected in the *c-Jun +* spots, and 314 DEGs in the *c-Jun-* spots (Figures 6I,J). GO enrichment analyses of all three IEG expressing spot populations across trained and untrained conditions revealed similar sets of enriched biological processes (Supplementary Figure 3D), namely those involved in ribosomal function and energy production. A finding which is consistent with the regionalized differences observed between the trained and untrained animals through spatial transcriptomics (Figure 4J).

In the analysis between *Egr1+* and *Egr-* spots, 555 DEGs were identified within the trained animal, and 196 DEGs within the untrained animal (Figures 6G,H). In the analysis between *c-Jun +* and *c-Jun*- spots, 480 DEGs were identified within the trained animal, and 166 DEGs within the untrained animal (Figures 6K,L).

Due to the high degree of correlation between the expression of *Arc* and *Egr1*, and the low correlation between the expression of *Arc* and *c-Jun*, we expected that *Arc* and *Egr1*-expressing spots would appear more functionally similar to one another than they would to *c-Jun*-expressing spots, within the same sample. Counterintuitively, comparisons of IEG+ and IEG- spots within each sample revealed distinct collections of biological processes overrepresented in the *Arc*, *Egr1*, and *c-Jun* expressing populations. Additionally, in contrast to the within sample analyses of *Arc* spots (Figure 5G), within sample analyses of *Egr1* and *c-Jun* spots were enriched with numerous biological processes in *both* trained and untrained mice. This effect is enhanced when enrichment analyses are stratified by up- and down-regulation. Biological processes enriched among DEGs in the *Egr1* spots within the trained and untrained mice were both involved in synaptic organization and plasticity (Figure 6M). Biological processes involved in RNA splicing, synaptic plasticity, and axonal growth were overrepresented in *c-Jun* spots in both trained and untrained mice. Together, these data suggest that IEG-expression following the memory retention test marks subsets of neurons which are enriched with distinct sets of biological processes.

## 4 Discussion

In this study, we used a combination of bulk RNA sequencing and spatial transcriptomics to identify changes in gene expression between APA-trained and untrained conditions, across each major subregion of the dorsal hippocampus and within IEG-expressing spots following memory recall elicited during retention test. A recent study utilizing spatial transcriptomics found distinct transcriptomic signatures in the hippocampal subregions following training in a spatial object recognition task (Vanrobaeys et al., 2023). This finding illustrates the power of using novel transcriptomic analyses to explore the spatial distribution of gene expression profiles when investigating memory-associated neuronal networks.

### 4.1 Regionalization of molecular mechanisms following memory recall

Different brain regions are recruited depending on the type of memory training an animal is exposed to Rolls (2000) and Tonegawa et al. (2018). We found evidence supporting the regionalization of gene expression in trained compared to untrained animals resulting in overrepresentation of GO terms across the dorsal hippocampus following spatial memory recall. A finding consistent with prior reports demonstrating enrichment of biological processes related to transcription and synaptic differentiation in the DG following APA recall (Harris et al., 2020). In our study, comparative bulk RNA sequencing analyses between trained and untrained animals revealed abundant overlap of detected DEGs and enriched biological processes between the CA1 and CA3 subregions. However, the transcriptional landscape observed in the DG exhibited divergence from both CA subregions, suggesting recruitment of specialized molecular pathways in the trained animals in a subfield specific manner following memory recall.

Cells in the CA1 and CA3 subregions share similar morphologies and ontology as compared to the dentate gyrus (Stanfield and Cowan, 1979; Khalaf-Nazzal and Francis, 2013; Cembrowski and Spruston, 2019), which could account for our regionalized findings in the bulk RNA sequencing analysis. During memory recall, the functional coupling of the CA3 and CA1 subregions (Montgomery and Buzsaki, 2007; Carr et al., 2012) is also reflected by the gene expression changes observed in this analysis. Our findings support the role of neurogenesis and protein synthesis in the DG (Alam et al., 2018; Kitamura et al., 2009; Kitamura and Inokuchi, 2014; Deng et al., 2010), as well as the memory-associated increases in efficacy in CA3-CA1 synaptic connections (Aksoy-Aksel and Manahan-Vaughan, 2013; Pavlowsky and Alarcon, 2012) associated with the APA training experience. Furthermore, our data also implicate enhanced energetic requirements in the DG during periods of high cognitive demand that enable accurate spatial navigation and memory recall (Mendez-Lopez et al., 2010; Christie and Schrater, 2015), evidenced by the enrichment of ATP metabolic pathways with the trained condition.

### 4.2 Technical considerations of using spatial transcriptomics

We employed an integrative approach combining bulk RNA sequencing and spatial transcriptomics to determine the transcriptomic profile elicited in the hippocampus following the recall of a consolidated spatial memory. Both methodologies provided unique utility and insight into the transcriptomic changes in the brain following memory and have proven to be highly compatible (Li and Wang, 2021; Vanrobaeys et al., 2023). Our bulk RNA sequencing uncovered robust hippocampal subregion-specific distinctions in biological process enrichment between trained and untrained conditions. Meanwhile, spatial transcriptomic analyses revealed anatomical stratification of DEGs but convergence on overlapping sets of enriched biological processes across the CA1, CA3, and DG cell layers. The apparent differences between the results obtained from these two results were unexpected. Between the two techniques, few of the regionalized DEGs were overlapping yet, we found that DEGs detected in each region were involved in the regulation of synaptic plasticity. This finding indicates that both techniques are detecting similar biologically significant changes supporting APA memory recall.

The distinct results noted by the bulk and spatial transcriptomic experiments potentially arise from key chemical and technical variances between the two methodologies. First, the 55 μm capture spots of the 10x Visium spatial transcriptomics platform permits a more granular analysis of gene expression differences between specifically annotated populations of cells, as opposed to the entirety of a bulk section (Stahl et al., 2016). Second, relative to bulk sequencing, 10x’s Visium spatial transcriptomics requires more tissue processing before RNA extraction and has lower RNA detection sensitivity (Asp et al., 2020), which might skew the genes detected between the two studies. Nevertheless, we surmise that our dual omics approach leverages the combined strength and resolution of these emerging technologies to explore the gene expression changes following spatial memory recall simultaneously within individual cell populations and across a spatially distributed neuronal network.

### 4.3 *Arc*-expressing spots are enriched with genes involved in synaptic plasticity

Using spatial transcriptomics, we examined the spatial distribution of IEG expression in the dorsal hippocampus to infer the location of neurons involved in memory formation. We hypothesized that spatially restricted subsets of IEG-expressing memory-associated neurons would be detected amongst these spots and enriched with genes related to synaptic plasticity. To test this, mice were sacrificed following retention test during the temporal window comprising the peak expression of IEGs as well as the initial upregulation phase of late response genes (Yap and Greenberg, 2018; Sheng and Greenberg, 1990). This approach enabled concurrent transcriptional profiling of both the rapid and delayed genomic responses linked to memory formation. The spatial patterning of IEG induction and overlap with plasticity-related genes could reveal the locations of neurons recruited to the memory trace with the dorsal hippocampus.

Comparisons of *Arc* + spots across the trained and untrained animal revealed enrichment of biological processes related to energy metabolism and ribosomal function. Critically, comparisons of *Arc* + versus *Arc*− spots within the trained animal were substantially different from those in the untrained animal. Specifically, *Arc* + spots in the trained animal displayed upregulation of biological processes related to synaptic plasticity, several of which were also enriched in the bulk analyses. These data imply that the significant functional modulation occurring selectively in the *Arc*-expressing neuronal population following learning may largely drive the transcriptional differences observed between training conditions in the analysis of bulk hippocampal subregions.

We found that the expression of *Arc* mRNA was only detectable in a subset of spatial transcriptomic spots in the hippocampus. This spatial distribution of *Arc* expression differs from reference atlases measuring the expression of *Arc* in the hippocampus through *in situ* hybridization (Kasukawa et al., 2011; Lein et al., 2007). This discrepancy can be accounted for by the lower RNA detection sensitivity of spatial transcriptomics, which would bias detectable *Arc* mRNA to the regions of space with the highest expression (Asp et al., 2020). As discussed previously, Arc expression is induced following robust memory-related neuronal activity (Ryan et al., 2015; Guan et al., 2016; Chen et al., 2019; Guzowski et al., 1999; Ramirez-Amaya et al., 2005; Lee et al., 2022). In our data we find the regions of space with the highest expression of *Arc* are mainly along the principal cell layer of the CA1 subregion. This finding indicates that this hippocampal subregion has the highest density of strongly activated *Arc +* neurons belonging to the memory associated neuronal ensemble.

10x Visium spatial transcriptomics permits a resolution of 55 μm, given the size of an individual capture spot. The density of cell bodies varies across regions of the hippocampus meaning that a spot of this size covers approximately 10–50 cell bodies and countless packed nerve fibers. Neuronal activity and IEG expression also vary by region and layer. In the densely packed granule cell layer of the DG, a small fraction of cells are active and strongly expressing IEGs (including Arc) after learning (Ramirez-Amaya et al., 2006). In contrast, the large pyramidal cells of the CA1 and CA3 principal cell layers are recruited in greater relative proportions following memory and have higher basal level of Arc expression (Guzowski et al., 2001). Therefore, the number of neighboring Arc-expressing cells required to reach a detectable level with spatial transcriptomics correspondingly varies by region. For example, the number of small granule cells required per capture spot might be greater than the number of required pyramidal cells. As such, we cannot determine the cellular composition of the spots (e.g., spots containing many cells expressing Arc versus spots containing one or few cells highly expressing Arc) across the regions of the hippocampus. Although the approach is not single-cell resolution, and multiple steps of the protocol decrease the sensitivity of the mRNA detection, we do reason that this spatial transcriptomic approach is suitable for the detection of highly expressed mRNAs, like the IEG mRNAs elicited by the reactivation of a memory. With discretion, we speculate that the training experience tunes molecular processes in cell populations (i.e., spatial spots), which we can define as microenvironments, to encode learned information.

We put forward the notion that the organization of the sparsely recruited Arc-expressing spots that we investigate in this paper may be constituted by subsets of memory engram cells neighbored by a network of complementary cells that creates a mesh of sinks and sources in neural activity (i.e., microenvironments) with the purpose of information coding. It is not yet known if the functional changes that distinguish cells in a memory-associated neuronal ensemble from surrounding cells are the same functional changes that distinguish these cells across training conditions. Our data suggest that there are two defining features of the *Arc +* spots: the exclusive upregulation of synaptic plasticity mechanisms relative to *Arc*- spots in the trained animal, and the upregulation of energy production and ribosomal function in the trained versus the untrained animal. In support of these findings, a recent study identified synaptic plasticity as a defining feature of a neuron in a memory-associated neuronal ensemble (Jeong et al., 2021). Additionally, increases in energy production and ribosomal biogenesis have been found during periods of high cognitive demand (Magistretti and Allaman, 2015; Hernández et al., 2015), which corresponds to the trained avoidance behavior of the APA paradigm (Cimadevilla et al., 2000; Wesierska et al., 2005).

### 4.4 *Egr1-* and *c-Jun*-expressing spots exhibit distinct transcriptomic profiles

Extensively studied for its role in memory storage, the Arc-tagged neuronal ensemble represents only a fraction of neurons activated during a memory event (Lacagnina et al., 2019; Sun et al., 2020; Sweis et al., 2021). Distinct subgroups of the neurons activated during a memory event are marked by several Immediate Early Genes (IEGs), which are associated with different patterns of intense neuronal activity (Okuno, 2011; Sun and Lin, 2016; Sheng et al., 1993; Guzowski et al., 2001; Tonegawa et al., 2018). This IEG expressing neuronal ensembles could encode multiple memory traces for a specific behavioral experience (Kitamura et al., 2017; Tonegawa et al., 2018; Tonegawa et al., 2015; Terranova et al., 2023). Using spatial transcriptomics, we assessed the gene expression profiles of spots expressing the IEGs *Egr1* and *c-Jun*, which were the IEGs most and least correlated with *Arc* expression, respectively.

*Arc+, Egr1+,* and *c-Jun +* spots displayed enrichment for largely distinct sets of biological processes within both trained and untrained animals. In contrast to the within sample comparisons of *Arc* spots, numerous biological processes were overrepresented within the trained and untrained animal for both *Egr1* and *c-Jun* spots. Specifically, *Egr1* spots displayed enrichment of biological processes related to synaptic organization and synaptic plasticity, whereas *c-Jun* spots displayed enrichment of biological processes related to RNA splicing, synaptic plasticity, and axonal projection. Additionally, the spatial distribution of *Arc* and *Egr1* shows high levels of expression in the CA1 cell layer of trained and untrained samples, while the expression of *c-Jun* is high in the DG. In one study, Egr1 protein was shown to have higher expression in the CA1 following 1 day old spatial memory retrieval, relative to later time points (Barry et al., 2016). In the same study, Arc protein was difficult to detect in the CA1 due to low staining contrast in trained and control animals. While our data replicated the finding for *Egr1* mRNA, they also point toward the utility of spatial transcriptomic studies to investigate the memory trace as it evolves over time.

Differing IEG induction likely relates to unique patterns of neuronal activation (Lyons and West, 2011; Madabhushi and Kim, 2018; Tyssowski et al., 2018; Flavell and Greenberg, 2008) and downstream circuit engagement (Roy et al., 2017), highlighting the functional and spatial diversity amongst recruited cells within memory associated neuronal ensembles. Both *Egr1* and *c-Jun* code for transcription factors whose downstream genes have been linked with synaptic plasticity and memory processes (Yelhekar et al., 2024). Genes downstream of Egr1-driven transcription are involved in vesicular release, neurotransmitter metabolism, receptor expression, and synaptic plasticity (Duclot and Kabbaj, 2017; Sekiya et al., 2019). Genes downstream of c-Jun-driven transcription in the brain are involved in neurite growth, axonal regeneration, and synaptic long term depression (Raivich and Behrens, 2006; Haeusgen et al., 2009). At the behavioral level, *Egr1* and *c-Jun* expression has been reported in response to fear memory and acute stress, respectively, while *Arc* expression has been linked to the encoding of contextual information (including location), in spatial memory tasks (Cheval et al., 2012; Maddox et al., 2011; Sherrin et al., 2010; Kubik et al., 2007; Gouty-Colomer et al., 2016; Cleland et al., 2017; Ramirez-Amaya et al., 2005).

It is well described that different patterns of neuronal activity can differentially trigger the expression of distinct IEGs (Okuno, 2011, Sun and Lin, 2016, Sheng et al., 1993, Guzowski et al., 2001, Tonegawa et al., 2018). And that different patterns of neuronal activity impinging on the same or distinct subsets of a neuronal population are also seen during the acquisition and retrieval of a memory experience (Lyons and West, 2011; He et al., 2019; Kim et al., 2018). While the direct link between types neuronal activity and IEG expression has yet to be established, it is plausible to model their connection as a continuous feature space that could describe cellular mechanisms across neuronal populations encoding of the various components of a memory experience (Kitamura et al., 2017; Tonegawa et al., 2018; Tonegawa et al., 2015; Terranova et al., 2023). In our study, the differences observed amongst the *Egr1*, *c-Jun,* and *Arc* expressing spots could be related to emotional, temporal, and spatial components inherent to both memory experiences of trained and untrained animals in the APA apparatus (Sun et al., 2020).

Presently, we cannot make specific connections between particular IEG-driven mechanisms and specific behavioral components of a memory experience. This remains a core gap in our knowledge of how the brain stores memory. Addressing this gap in knowledge would necessitate an approach linking molecular mechanisms and neuronal networks to simultaneously identify biological pathways associated with behavioral features (e.g., aversive or rewarding, early-term or long-term), the subset of neurons enhancing/repressing those pathways and, importantly, the connectivity of these neurons (inputs and outputs) within brain circuits.

### 4.5 Investigating memory across multiple scales in the brain

In summary, we performed an integrated investigation of hippocampal transcriptional dynamics following spatial memory recall leveraging the high sensitivity bulk RNA sequencing to complement the high spatial resolution of spatial transcriptomics. Regionally restricted expression patterns were observed with the CA1 and CA3 subregion exhibiting enrichment for synaptic plasticity and transmission pathways, while the DG was more prominently categorized by enrichment for protein synthesis and energy metabolism pathways. We identified a specialized signature of *Arc* expressing spots in trained mice categorized by upregulation of genes involved synaptic plasticity and transmission. Additionally, functionally distinct IEG expressing populations were revealed with *Arc, Egr1,* and *c-Jun* expressing spots exhibiting differential pathway enrichment and anatomical distribution.

The hippocampal transcriptional landscape captured in this study of APA memory recall represents a single transitionary state over the life of a memory. Networks in the hippocampus undergo complex spatio-temporal tuning in flow of information depending on memory phase, valence, and cognitive load (Terranova et al., 2022; Roy et al., 2017; Marks et al., 2022). As a memory evolves through systems consolidation, the neural ensembles, cellular properties, and molecular profiles supporting the memory trace are thought to transform correspondingly (Tonegawa et al., 2018; Alberini and Kandel, 2014). However, capturing this gradual reshaping of memory representations across hippocampal subregions has remained challenging. Emerging spatial molecular profiling techniques offer unmatched resolution for mapping distributed neuronal populations while preserving native tissue context. Looking forward, studies exploring the molecular memory trace would benefit from employing multiple transcripomic technologies on the same neuronal system to gather vital information across spatial scales, as we have done here. By applying transcriptomic methods to concurrently inspect spatial loci and neuronal populations, the topological features of the molecular signatures supporting memory could be elucidated.

## Data Availability

The data for this project can be found using accession numbers GSE279272 and GSE279273. GSE....72 has the microdissection experiments data. GSE...73 has the data for the spatial transcriptomic experiments. They can be found published and released at the following links: https://www.ncbi.nlm.nih.gov/geo/query/acc.cgi?acc=GSE279273, https://www.ncbi.nlm.nih.gov/geo/query/acc.cgi?acc=GSE279272.
